# Supplementary material for: Men’s education and intimate partner violence—Beyond the victim-oriented perspective: Evidence from demographic and health surveys in Central Africa
Source: PLoS One. 2024 Apr 25;19(4):e0302627. doi: 10.1371/journal.pone.0302627 (PMC11045122; doi:10.1371/journal.pone.0302627)
Supplement: Attachment — Submitted filename: Point-by-point responses .docx [file pone.0302627.s001.docx]

**Point-by-point responses to reviewers’ comments and suggestions.**

**Manuscript: PONE-D-23-33735**

Title:

**Men’s education and intimate partner violence—Beyond the victim-oriented perspective: Evidence from Demographic and Health Surveys in Central Africa**

**Reviewer #1**

The manuscript should include a section on limitations and, perhaps, directions for future research. The limitations section should be detailed and thorough.

**Response: The authors do appreciate this comments. A section on Study limitations (see p. 31) has been included in the revised version of the manuscript. As per directions for future research, this was already suggested in terms of “policy implications” on “interventions to equalize levels of education between spouses” since the gap in education among spouses could be harmful for women especially if these latter are more educated.**

I suggest moving most of the section's “analytical strategy” content to an appendix while keeping the descriptive analysis paragraph and including a brief description and reference to the appendix.

**Response: The authors took this comment very positively. However, this does not comply with PLOS One requirements since materials in appendix fall under “not reviewed”. In a paper including modeling/estimations, analytical strategy is of chief importance. In addition, it is approximately one-pager short enough not to be a viable “appendix”.**

**Reviewer #2**

- Some sentences in the introduction need clarification

**Response: The authors effortlessly read/edited the introduction to ensure that all sentences are clear for the readers.**

- The manuscript needs to enrich the theoretical framework

**Response: The authors did efforts to ensure that theoretical framework is enriched. However, the reviewers should also note that due to limited space, we couldn’t extend indefinitely the theoretical framework to keep the manuscript concise, both theoretically and empirically. To do that, each theory was succinctly presently; followed by the evidence generated so far.**

-Show the limitations of the study better

**Response: The authors do appreciate this comments. A section on Study limitations (see p. 31) has been included in the revised version of the manuscript.**

- Study procedures are good

**Response: Thanks; we do appreciate it.**

- References are up to date

**Response: Thanks; we do appreciate it.**
